# Social Support Predicts Depressive Symptoms in Dementia – A Four‐Year Longitudinal Study

**DOI:** 10.1002/alz70858_102810

**Published:** 2025-12-25

**Authors:** Lina Charlotte Jeran, Nadia Blecha, Francisca S Rodriguez, Bernhard Michalowsky, Stefan Teipel, Wolfgang Hoffmann, Jochen René Thyrian, Iris Blotenberg

**Affiliations:** ^1^ Deutsches Zentrum für Neurodegenerative Erkrankungen e. V. (DZNE), site Rostock / Greifswald, Greifswald, Mecklenburg‐Vorpommern, Germany; ^2^ Department of Psychosomatic Medicine, University Hospital Rostock, Rostock, Mecklenburg‐Vorpommern, Germany; ^3^ Deutsches Zentrum für Neurodegenerative Erkrankungen e. V. (DZNE), site Rostock / Greifswald, Rostock, Mecklenburg‐Vorpommern, Germany; ^4^ Institute for Community Medicine, University Medicine Greifswald, Greifswald, Mecklenburg‐Vorpommern, Germany

## Abstract

**Background:**

Depressive symptoms are a common neuropsychological symptom in people with dementia. They are associated with reduced well‐being and may exacerbate dementia symptoms. So far, there has been little research on how modifiable factors, such as the social environment, are associated with the severity of symptoms. The aim of the present study was to investigate the role of support from the social environment for depressive symptoms in community‐dwelling people with dementia – beyond sociodemographic and clinical factors.

**Method:**

We used data from 378 people screened positive for dementia in primary care (Mage = 80.2, 59.5% female) who were interviewed annually in their homes by specially qualified nurses. Social support was assessed using the Questionnaire for the Assessment of Social Support (FSozU), depressive symptoms were measured using the Geriatric Depression Scale (GDS). We used multilevel growth curve models with random intercepts and slopes to model depressive symptoms over time. We modelled both the role of between‐person differences and the role of within‐person changes in social support for depressive symptoms.

**Result:**

At the beginning of the study, the number of people with dementia who reported mild to severe depressive symptoms was 15.6 %. More social support was associated with fewer depressive symptoms overall over the four‐year period (blog = ‐0.27, % change: ‐23.66, 95% CI: ‐32.29, ‐13.93). A decline in social support was associated with more depressive symptoms (blog = ‐0.23, % change: ‐20.55, 95% CI: ‐28.82, ‐12.19). These effects remained stable after controlling for sociodemographic and clinical factors.

**Conclusion:**

This is the first study to show that the social environment plays an important role in depressive symptoms in people with dementia – above and beyond clinical factors. Social support as a modifiable factor may be a lever for alleviating depressive symptoms in dementia. In the care of people with dementia, not only medical but also psychosocial needs should be given greater attention.

